# Engineered extracellular vesicles as “supply vehicles” to alleviate type 1 diabetes

**DOI:** 10.20517/evcna.2024.61

**Published:** 2024-11-14

**Authors:** Fei Wang, Zhenhua Li

**Affiliations:** ^1^The Tenth Affiliated Hospital, Southern Medical University (Dongguan People’s Hospital), Dongguan 523059, Guangdong, China.; ^2^Guangdong Provincial Key Laboratory of Cardiac Function and Microcirculation, Southern Medical University, Guangzhou 510515, Guangdong, China.

**Keywords:** Extracellular vesicles, type 1 diabetes, PD-L1, Gal-9, hyperglycemia

## Abstract

Recent findings have indicated that the deficiency of inhibitory programmed cell death ligand 1 (PD-L1) and galectin-9 (Gal-9) in pancreatic β-cells is associated with the progression of type 1 diabetes (T1D). This suggests that exogenous PD-L1 and Gal-9 may have promising potential as therapeutics for the treatment of T1D. In light of these reports, a recent work investigated the potential of artificial extracellular vesicles (aEVs) with the presentation of PD-L1 and Gal-9 ligands (PD-L1–Gal-9 aEVs) as a treatment for T1D, with the findings published in *Diabetes*. Notably, the PD-L1–Gal-9 aEVs demonstrated the capacity to induce apoptosis of T cells and the formation of regulatory T (Treg) cells, thereby maintaining immune tolerance. Furthermore, the *in vivo* administration of PD-L1–Gal-9 aEVs resulted in a reduction in T cell infiltration in the pancreas, an increase in β-cell integrity protection, a significant decrease in blood glucose levels, and a delay in the progression of T1D. In conclusion, this study proposed an innovative approach to the treatment of T1D progression through the use of immunosuppressive EVs. This highlight provides a comprehensive analysis and discussion of the pivotal findings of this study.

## MAIN TEXT

Type 1 diabetes (T1D) is an autoimmune disease that is characterized by elevated blood glucose levels. This results from T lymphocyte-caused damage to the β-cells in the pancreas and subsequent insulin secretion failure^[1,2]^. T1D accounts for 5%-10% of all reported diabetic cases, with a globally increasing incidence^[3]^. It is postulated that T1D is a highly heterogeneous disease, influenced by a variety of factors, including age, genetic alterations, and environmental influences. Research suggests that pancreatic β-cells and their interactions with immune cells play a pivotal role in the development and pathogenic progression of T1D^[4]^. Insulin therapy is considered the primary treatment option for T1D, as it has a crucial role in the regulation of glucose intracellular transportation^[5-7]^. However, insulin therapy is confronted with significant adverse effects, including hypoglycemia and insulin resistance. Considering the interactions between pancreatic β-cells and immune cells, a variety of immunosuppressive therapies have been proposed as potential alternatives for the treatment of T1D^[8]^. In 2022, the US Food and Drug Administration (FDA) approved teplizumab, a CD3-binding monoclonal antibody, for the prevention of T1D. Nevertheless, teplizumab therapy may result in severe side effects and is currently expensive^[9]^. Therefore, it is necessary to develop treatment methodologies with enhanced therapeutic effects for the prevention and treatment of T1D.

Recently, nanotechnology-based biomaterials have been employed extensively as therapeutic agents or innovative drug delivery platforms in treating a variety of illnesses, including T1D^[10-13]^. Among the various types of nanomaterials, extracellular vesicles (EVs), which are nano-sized phospholipid bilayer membrane-enclosed particles secreted by various cell types, have attracted considerable interest for their potential as a next-generation nanomedicine with applications in numerous biomedical fields^[14-18]^. In particular, it has been demonstrated that EVs derived from various cell resources, including mesenchymal stem cells (MSCs), adipocytes, and endothelial progenitor cells (EPCs), achieved remarkable therapeutic efficacy in the treatment of T1D^[19]^. For instance, MSC-derived exosomes have been demonstrated to stimulate the regeneration of pancreatic islet cells and insulin secretion by regulating a group of genes^[20]^. In addition, microvesicles derived from EPCs were shown to facilitate the neoangiogenesis process of the transplanted islet via the delivery of miRNAs^[21]^.

It is noteworthy that EVs can be utilized as drug delivery platforms for various biomolecules, such as nucleic acids, proteins, and chemotherapeutic drugs^[22-25]^. For example, Wang *et al*. proposed an inhalable vaccine for the prevention of SARS-CoV-2^[26]^. The vaccine is developed using lung-derived exosomes with conjugation to the recombinant domains binding to SARS-CoV-2 receptors (RBD). The exosome vaccine was observed to improve the retention rate and time of RBD within the lung, and also inducing systemic mucosal immunity and T cell immunity in mice and hamsters^[26,27]^. In another report, decoy exosomes modified with GC-enriched tetrahedral DNA nanostructures have been shown to mitigate doxorubicin (DOX)-induced hepatotoxicity by scavenging DOX and polarizing macrophages into the M2 phenotype^[28]^.

Given their remarkable capacity to carry cargo, EVs could also be employed as “supply vehicles” of therapeutic biomolecules in the treatment of T1D. A deficiency in immune checkpoint molecules with pro-inhibitory effects, including programmed cell death ligand 1 (PD-L1) and galectin-9 (Gal-9), has been observed in pancreatic β-cells and has been linked to the progression of T1D^[29]^. Accordingly, Yang *et al.* postulated that the delivery of exogenous PD-L1 and Gal-9 to β-cells might result in an immunosuppressive therapeutic effect, thereby alleviating T1D^[30]^. By leveraging genetic engineering technology, the authors generated Raw264.7 cells overexpressing PD-L1 and Gal-9 molecules on their cell membranes. The overexpression of Gal-9 resulted in the transformation of the Raw264.7 cells to an M2 macrophage phenotype. In addition, genetically engineered Raw264.7 cells were demonstrated to generate artificial extracellular vesicles (aEVs) with high expression levels of PD-L1 and Gal-9 (called PD-L1–Gal-9 aEVs). The developed EV-based nanoplatforms displayed the binding ability to the PD-1 and T-cell immunoglobulin mucin 3 (TIM-3) receptors on the T lymphocytes. The binding is attributed to the interactions between PD-L1 and PD-1, as well as Gal-9 and TIM-3.

The authors showed that the PD-L1–Gal-9 aEVs induced a significant inhibition of the AKT signaling pathway and the induction of T cell apoptosis. Treatment with PD-L1–Gal-9 aEVs resulted in notable expansion of the regulatory T (Treg) cell population. Furthermore, this study provided *in vivo* evidence of the therapeutic effects of the developed EV-based nanoplatforms. *In vivo* imaging results demonstrated the distribution of the developed EV-based nanoplatforms in the pancreas following intravenous administration. Moreover, the treatment with PD-L1–Gal-9 aEVs was observed to markedly attenuate T1D progression in a diabetic mouse model, accompanied by elevated levels of C-peptide and IL-10 expression. Additionally, PD-L1–Gal-9 aEVs demonstrated capacity in safeguarding pancreatic β-cells from destruction, impeding the T cell infiltration, and enhancing the population of Treg cells within the pancreatic tissue.

The study collectively presented an aEV-based platform for the delivery of therapeutic factors to pancreatic tissue, with the objective of improving T1D outcomes. The destruction of β-cells in the pancreas tissue by autoreactive T cells represents a critical cause of T1D. The authors demonstrated that the administration of immunosuppressive PD-L1 and Gal-9 represents an efficacious approach to inhibit the infiltration of T cells and alleviate T1D. This study suggested that aEVs could serve as a delivery system for macromolecules, such as proteins, for the treatment of T1D. In light of the benefits associated with aEVs, including high yield, reduced expense, and fewer side effects, aEVs are emerging as a promising therapeutic nanoplatform for the treatment of T1D.

## DECLARATIONS

### Copyright

© The Author(s) 2024.
